# Prioritising patient-centredness and service equity for long-term survivors of BMT: a qualitative study of healthcare professionals

**DOI:** 10.1007/s00520-025-09577-5

**Published:** 2025-06-05

**Authors:** Gemma McErlean, Christine Ashley, Anisha Pradhan, Vanessa Yenson, Ian Kerridge, Elizabeth Halcomb

**Affiliations:** 1 School of Nursing, Faculty of Science, Medicine & Health, University of Wollongong, Wollongong, NSW Australia; 2 Health Innovations, Faculty of Science, Medicine & Health, University of Wollongong, Wollongong, NSW Australia; 3 Centre for Research in Nursing and Health, St George Hospital, Kogarah, NSW Australia; 4 Faculty of Health, University of Technology Sydney, Sydney, NSW Australia; 5 Royal North Shore Hospital, Sydney, NSW Australia; 6 Sydney Health Ethics, University of Sydney, Sydney, NSW Australia

**Keywords:** Bone and marrow transplant, Prioritising care, Equity, Patient centred, Long-term survivors, Telehealth, Shared care models

## Abstract

**Purpose:**

Survivors of blood and marrow transplant (BMT) for malignancies experience significant morbidity and mortality resulting from the long-term and late effects of transplant, necessitating life-long care. The purpose of this study was to explore the perceptions of specialist BMT advanced practice nurses (APNs), physicians, and primary care practitioners regarding the challenges of delivering long-term care for survivors of BMT.

**Methods:**

This descriptive qualitative study used semi-structured interviews with 13 purposefully selected healthcare professionals (HCPs) experienced in providing long-term care to survivors of BMT. Data were analysed using thematic analysis.

**Results:**

Two themes were identified: (1) prioritising patient-centred care and (2) equity and access to services. Prioritising patient-centred care included the sub-themes of (a) the burden of survivorship and complexity of long-term care, (b) education and preparation for long-term follow-up, and (c) adherence with long-term follow-up care. Equity and access included the four sub-themes of, (a) the tyranny of distance, (b) the price of survival, (c) primary care and the availability of GPs, and (d) access to appropriate long-term care.

**Conclusions:**

HCPs need to adopt patient-centred strategies to improve optimal care and equity for long-term BMT survivors. Practical approaches include providing comprehensive education and personalised care, performing routine needs assessments, implementing flexible models of care that integrate primary care such as shared care and telehealth. Using digital health platforms and advocating for reduced financial barriers can further address challenges in access and equity. These efforts have the potential to enhance long-term care delivery, improve outcomes, and ultimately enhance quality of life for BMT survivors.

## Introduction

Blood and marrow transplantation (BMT) provides optimal care for adults and children with a range of malignant and non-malignant disorders [1]. With improvements in therapy, supportive care, donor selection, and quality management, the number of people undergoing this life-saving procedure is increasing annually, with growing numbers of recipients surviving long-term [1, 2]. However, this success brings significant long-term morbidity and mortality, requiring lifelong monitoring and preventive care [3].

International guidelines support the provision of high-quality lifelong care for survivors of BMT to optimise health outcomes [4, 5]. However, adherence to these guidelines is complex and demanding, requiring survivors to engage with multiple health professionals annually for up to 34 different assessments including clinical examinations, blood tests, diagnostic imaging, psychosocial, and lifestyle risk assessment [4, 6, 7]. As the BMT survivor population ages, there is also increasing need for care strategies that address the specific healthcare needs of adult survivors of childhood BMT, older adults, and those with co-morbidity [8].

In Australia, the delivery of long-term care for BMT survivors mirrors international practices [5, 6], with enormous variation in follow-up care models influenced by patient demographics, survivor case-loads, resource availability, workforce capacity, and health system strengths [5]. Follow-up is particularly challenging for survivors in rural and remote areas who must travel long distances to major centres. This challenge is exacerbated by factors such as social isolation [9], educational disruptions, and employment challenges due to illness and healthcare demands [10]. Consequently, BMT survivors often receive fragmented care across tertiary and community-based public and private services [11], leading to financial, psychological, and social burdens for patients and their carers, complicating health professional relationships.

Experts in post-BMT care, including Hewitt et al. [12], advocate for comprehensive cancer survivor care characterised by safety, effectiveness, patient-centredness, timeliness, efficiency, and equity. Despite the existence of national and international guidelines for long-term post-BMT care [5], there is limited understanding of how well current practices align with these principles or the perspectives of healthcare professionals regarding the delivery of long-term BMT care [2, 4, 10]. This study aims to address this knowledge gap, with a focus on integrating primary care to improve outcomes for cancer survivors.

## Methods

### Study design

This study employed a descriptive qualitative design with semi-structured interviews of clinicians experienced in caring for BMT survivors. This methodology provided a comprehensive examination of participants’ experiences and perspectives. The Consolidated Criteria for Reporting Qualitative Studies (COREQ) checklist was used to guide reporting [13].

The interviews explored health professionals’ perceptions, identified barriers and facilitators to providing long-term follow-up (LTFU) care, and examined optimal care models. This paper focusses on the challenges of delivering long-term care for survivors of BMT, while data on models of care delivery is published separately [14].

### Participants

Specialist Haematologists, General Practitioners (GPs), and Advanced Practice Nurses (APNs) registered with their respective professional board possessing recent experience in providing care to long-term survivors of BMT were included in this study. Health professionals whose experience was limited to inpatient care during the pre- or early post-BMT period were excluded.

### Recruitment

Purposive and snowball sampling was used to ensure diverse geographical representation, targeting participants from metropolitan, regional/rural areas. Two recruitment strategies were used:

1. Targeted recruitment: GPs were identified from BMT databases at two New South Wales haematology departments. Practice managers were contacts, and study packs (including participant information sheets and consent forms) were sent to interested parties.
2. Broad recruitment: Study information was disseminated through primary health networks, professional organisations, and social media platforms, targeting GPs, APNs, and specialist haematologists. Interested health professionals contacted researchers directly for study packs.

Interviews were scheduled at convenient times for consenting participants, who were assured of their right to withdraw at any time and the confidentiality of their input.

### Data collection

Two research team members conducted individual semi-structured telephone interviews. Each session was digitally recorded and transcribed verbatim to ensure accuracy and facilitate analysis. The number of interviews was determined by the principles of data saturation and information power. Data saturation is defined as the point at which no new information, themes, or insights relevant to the research questions emerged from subsequent interviews [15]. While information power suggests that the more relevant information the sample holds for the study aim, the fewer participants are needed. Factors such as the specificity of the sample, the clarity of the study aim, the use of established theory, the quality of dialogue, and the chosen analysis strategy determine information power [16]. Interviews were analysed iteratively as they were conducted, and continuously appraised regarding their information power.

Recruitment and interviewing ceased once no new themes emerged, and it was agreed by the study team that the richness and relevance of the data had addressed the study aims [16].

### Data analysis

Data were analysed using thematic analysis. Based on Braun and Clarke’s approach [17], this approach includes familiarisation with the data, generation of initial codes, search for themes, review of themes, definition and naming of themes, and production of the report. Analysis began immediately after the first interview, allowing for continuous refinement of questions to explore emerging themes in subsequent interviews. To protect participant privacy, study numbers were assigned, and locations and workplaces were redacted. Transcripts were imported into NVivo™ (2012 version) for analysis. To enhance reliability, three team members (GM, CA, EH) independently coded the data. Each coder initially reviewed and coded three transcripts separately, identifying preliminary codes and potential themes. Following this independent coding, the team met to compare their coding, discuss interpretations, and collaboratively refine the initial set of themes. This process of revision and review continued iteratively after three additional interviews had been completed until consensus was reached on the final thematic structure and no new themes were emerging. The iterative nature of this approach ensured our diverse perspectives were integrated and individual bias was minimised, resulting in a consistently applied coding framework across the dataset.

During theme development, the team engaged in open discussion to resolve any differences in coding or interpretation. Where disagreements arose, these were addressed through dialogue and consensus-building. The process of theme refinement involved reviewing all coded data extracts, grouping codes into broader themes, and ensuring that each theme was distinct, coherent, and well-supported by the data.

### Ethics

The Human Research Ethics Committee of the South Western Sydney Local Health District (Approval No. 2022/ETH01503) approved this project. All participants provided informed consent, and the research was conducted in accordance with the Declaration of Helsinki.

### Rigour

The study’s rigor was ensured by adhering to Lincoln and Guba’s [18] criteria: credibility, dependability, confirmability, and transferability. To enhance credibility, at least two researchers (GM, CA) independently reviewed all transcripts, reaching consensus at each stage of the study. Dependability was maintained through meticulous documentation of all study-related decisions and regular team meetings to discuss the research progress. Confirmability was addressed by maintaining a clear audit trail of the research process. To support transferability, the study provided detailed contextual information and rich descriptions of findings, allowing readers to evaluate their applicability to other settings.

## Results

Thirteen participants were interviewed; this included six (46.1%) APNs, four (30.8%) BMT specialists, and three (23.1%) GPs. Interview durations ranged from 21 to 60 minutes (mean 36 minutes). Two themes were identified in the data regarding the challenges of delivering long-term care for survivors of BMT, namely: prioritising patient-centred care and equity and access to services.

1. Prioritising patient-centred care
  Prioritising patient-centred care comprised three sub-themes: (a) the burden of survivorship and complexity of long-term care, (b) education and preparation for long-term follow-up, and (c) adherence with long-term follow-up care.
  - The burden of survivorship and complexity of long-term care
    All participants noted the importance of long-term follow-up after BMT while recognising the burden this placed upon BMT survivors and their carers. Participants noted that effective long-term follow-up required a multidisciplinary approach involving various specialties appropriate to the evolving needs of survivors throughout their post-transplant journey.
    … the long-term follow-up. This is a crowded room, right, so it’s not just a doctor and a nurse practitioner, it’s all the other people in the room, right, it’s the patient and the GP, or the community nurse practitioner or what have you. (BMT Specialist 3)
    Beyond the requirements from the health care team, participants expressed the importance of an engaged and activated patient.
    The patient has to know what’s required of them and has to be keen to do it. (BMT Specialist 3)
    The patient should be involved in his.. or her own care. That’s very important. (GP 1)
    Participants consistently emphasised the diverse range of long-term complications faced by BMT survivors and highlighted the necessity for life-long, multidisciplinary care. They stressed the importance of appropriately monitoring and managing physical, psychological, and social challenges over the long term.
    We don’t discharge transplant patients. They’re a patient for life… certain medical conditions, metabolic syndrome, skin cancers. They’re basically at higher risk of those things even many years down the track. (BMT Specialist 1)
    The other thing is just looking at involving more non-medical people… whatever is needed, according to the patient, whether they need any physical therapy, they need any psychological follow-up and treatment. (GP 1)
    Several participants described how the specific components and delivery of long-term follow-up could vary enormously between survivors, with those perceived to be at high risk of increased mortality and morbidity requiring closer monitoring. A patient-centred care approach was identified as being key to managing this care and improving the patient experience. There was also emphasis on a coordinated effort from various healthcare professionals to address the complex and evolving needs of survivors throughout their post-transplant journey.
    I guess you know which ones to keep; the ones that are profoundly immunosuppressed, or the ones that have significant organ dysfunction.... I think it can be tailored to the patient. (BMT Specialist 4)
    There’s a lot of patient-centred care like I do. I spend a lot of time on the phone, and I generally do oversee the appointments which is a massive administrative burden. But to ensure that it flows… I take it on. Because there’s nothing that I can’t stand more than a patient’s [care] like, ‘They’ve got gynae last Thursday, they’ve got the doctor this Thursday, and then they’ve got the respiratory the following [week].’ (APN 1)
    But this approach was dependent on effective navigation and communication in providing patient-centred care.
    I sit there and think, come on guys, we just need to pick up a phone, coordinate this. Because it’s about the patient and their experience. They are tired, they are really tired. So I generally say that to patients. I say, ‘Look, if the appointments aren’t working out, you’re getting frustrated, give me a call.’ (APN 1)
    The importance of listening to and respecting the views of the patient in terms of their preferred means of receiving long-term follow-up was also raised by participants, who described how BMT survivors frequently needed reassurance and ongoing reminders of the importance of long-term follow-up, and the differing roles of their transplant specialists and community practitioners:
    And then, as you say, post-transplant, it’s like well what do I do now? I’ve had my transplant, I’ve finished my treatment, now what do I do?. (APN 5)
    We can say at this point you’ll be given an option of going to principally a GP, but we will still ask you to come back every two years and then subsequently every five years, we will still do that…. Or alternatively, say, look, you’ll be seeing your GP for all of these things, and you’ll be seeing your local haematologist for all of these things... So, I think it can be made more patient centred as well as more standardised and harmonised and efficient. I think these things are actually all possible, they just require some proper thinking. (BMT Specialist 3)
    Some participants noted that not all health professionals took a patient-centred approach however, with priority sometimes given to organisational convenience rather than patient needs. This observation highlights a potential disconnect between the ideal of patient-centred care and its practical implementation in busy clinical settings:
    I don’t think the focus is patient outcome, patient centred…. Doctors get annoyed they don’t have the blood [results available]. And it’s like, ‘Well, it’s less about you having your blood tests [results] than the convenience for the patient.’ (APN 1)
    Several participants described how when communication with patients worked well, unique relationships developed over time, enhancing the quality of long-term follow-up:
    I think the patients actually – they build up these relationships with their doctor over a period of years and they have a lot of trust… it’s that relationship – that sort of lifesaving thing that they’ve gone through really makes those people and the teams so important to them. (BMT Specialist 1)
  - Education and preparation for long-term follow-up
    Empowering patients to become involved in their long-term care through education and support was identified as an important part of long-term follow-up. However, many participants recognised significant challenges in delivering this education effectively. BMT Specialist participants highlighted the difficulty of providing comprehensive information about long-term follow-up during pre-BMT discussions.
    Most people going into transplant would not necessarily have a vision for what their life will look like five years down the track. (BMT Specialist 1)
    When I’m seeing them in the months post-transplant I will talk about this concept of long-term follow-up, so it’s probably sort of a few months after their transplant.... Because mostly in the lead up to the transplant they are so overwhelmed with other information about the actual process – the transplant process itself and the risks… (BMT Specialist 2)
    GPs also commented on the need for survivors to understand both BMT-specific and general health risks:
    .. They need to understand the complications obviously and understand what complication symptoms are so they can go to emergency or to GP or to their, call their specialist or the team early. Yeah, that’s about the BMT. And then they need to know about the other medical conditions that can affect the long-term survival of anything like diabetes, cholesterol, high blood pressure, ischemic heart disease, obesity, they need to understand those also play a part in the survival and the quality of life after BMT. (GP 1)
    Participants also noted significant difficulty in providing structured and standardised education due to the variable nature of long-term and late effects.
    …long-term follow-up is much less protocol driven... it’s much more dependent on a multitude of different factors that can change.… (APN 3)
    Some participants observed that patients often lacked awareness of the importance of long-term follow-up.
    They’re not even aware that the long-term follow-up is obviously as important as what you see it to be. And it’s funny after all the work that’s been done in long-term follow-up and we’ve created guidelines and all this. The patients aren’t actually aware of it as much as we think they are. (APN 4)
    Additionally, not all survivors were seen to be receptive to education, with factors such as health literacy and language barriers influencing their understanding of their risks and need for long-term follow-up.
    You can still do a lot of education and a lot of guidance, and you still have those few that just don’t, maybe their health is not a priority, or education, literacy, it’s just easier just to call [the clinician] than actually process what they’ve been told. They get into a flap, and they ring. And it’s like, ‘Well, what did we talk about?’ …But it’s hard, it’s hard, because I also know there’s a lot of cognitive changes and psychosocial… I think is huge. (APN 1)
    I think the barriers are, language is probably one of the biggest. Language and actual understanding…Yeah, like health literacy. Because there’s some patients that you can explain everything to them 10 times and they just don’t get it, they just don’t understand. So there are barriers, but I also think it could be done better. (APN 5)
    Participants also noted how some survivors appeared reluctant to educate themselves due to fear of knowing what might happen.
    Sometimes I’ve given the pack and they’ll come to the education, and they’ll be like, ‘I’ve not even looked at it.’ And I’m like, ‘That’s fine…’ Or I’ve given the pack to them and they’re like, ‘I’m not ready to read it.’ And I’m like, ‘That’s OK.’ … because the book goes through everything and some people come back and be like, ‘Oh my God, that book really scared me.’ (APN 5)
  - Adherence to long-term follow-up
    Adherence to long-term follow-up care emerged as a significant challenge, with a complex interplay of factors influencing patients’ engagement with ongoing care. While participants reported that most survivors engaged with follow-up schedules, some survivors were noted to struggle with adherence, either due to personal circumstances or choices or to structural barriers. As BMT Specialist 2 noted, “the main barrier is patient choice and [the] patient’s decision to come back over many years. But as I said, most patients have no problem with that. …So we do have a few patients who have stopped coming, but we do try to chase them up.”
    Patient autonomy emerged as a significant challenge to adherence, with participants describing how some survivors actively decide to discontinue follow-up care over time despite healthcare providers’ persistent efforts to emphasise its importance. BMT Specialist 3 explained, “I mean the people that tend to come to see me are medically literate and already vigilant. Because the ones that don’t come are the ones who either don’t really realise that they probably should be doing it, or haven’t – or have disengaged from this. Some people are so traumatised by the whole situation that they never want to come back. Some people are so well that they don’t see the point in coming back.” When survivors made such decisions, the participants felt that this was because of the survivor’s perception of their health status and need for medical surveillance, their emotional readiness to (re)engage with medical care, and their life circumstances.
    Healthcare providers expressed frustration with the challenges of maintaining contact with “disengaged” patients over the long-term. A number of participants described how some survivors remained disengaged from post-BMT healthcare despite multiple efforts to engage them, including repeated phone calls and written communications. For many participants, this pattern of non-adherence not only complicates the delivery of comprehensive care but also raises concerns about the long-term health outcomes for these survivors.
    …there’s a few patients over the years who have just become lost because they don’t show up to their appointment with me. I usually try and ring them at least three or four times. – people have said, ‘Oh yes, I’m really sorry, make me another appointment, I’ll come again.’ And then they don’t show again. And then they don’t show again after that. And it gets to a point where I can’t just keep chasing…. There have been a few occasions where I’ve just had to send [a] letter to their GP saying, ‘Look, this person’s failed to turn up to appointments. We can’t keep offering them. And that we would be more than happy to see them again if they wanted to come.’ (APN 6)
2. Equity and access to services
  All participants described factors relating to equity and access to services as potential barriers to providing best-practice long-term follow-up to BMT survivors. The theme of equity and access to services included the subthemes of (a) the tyranny of distance, (b) the price of survival, (c) community care and the availability of GPs, and (d) access to appropriate long-term care.
  - The tyranny of distance
    Access to specialist haematology departments for long-term follow-up was identified as a significant challenge, particularly for patients living in rural or regional areas or those with caregiver responsibilities themselves. Travelling long distances to metropolitan centres was described as both physically and financially taxing. It’s “a lot of travel, it’s often very expensive for the person in terms of travel, accommodation, private specialist appointments” (GP 2). APN 3 agreed that “it’s a bit of a trip for the patients to be coming all the way here.” In particular, GP 1 stated: “They go there and they’ve got a 15-min appointment, and then they’ve got a two-hour journey back.” The logistical challenges of travel were seen to discourage patients from attending follow-up appointments. BMT Specialist 1 remarked, “I think if they need to travel they don’t come, because it’s not really worth their while.”
    Participants discussed the potential for local healthcare providers to alleviate some of these travel burdens. By involving GPs in monitoring and care, unnecessary trips to distant specialist centres could be avoided.
    If it’s a simple situation, relatively uncomplicated, then yes it would be very reasonable in some circumstances for a GP to do that. They don’t need to come here to do it. (BMT Specialist 1)
    I think it’s about...hospital centres knowing what is possible in general practice as well, or other primary care, psychologists and all the other allied health as well that’s very local for that person, and perhaps more affordable and geographically accessible. (GP 2)
  - The price of survival
    Economic inequities resulting from the financial burdens associated with long-term follow-up care for BMT survivors were identified as significant barriers to the provision of optimal healthcare. Beyond the costs of travel, participants highlighted several financial challenges that can deter survivors from seeking necessary care, including costs for consultations, tests, and vaccinations.
    Lack of reimbursement for health serviced provided in primary care settings that were provided free of charge by hospitals created significant financial and structural challenges for survivors. Some participants described how they addressed these inequities for the benefit of their patients/survivors:
    ..sometimes I’ve just faxed the request forms to where they have to go locally, so that they’re not out of pocket to some extent.… Or we might have to go private, and we just see if they can get bulk billed. (APN 6)
    So we started partnerships with external providers… [they gave access] to our laboratories that are in all the other hospitals. So I made a commitment to them and said, ‘Right, if they’re near one of those [public] facilities I’ll get them to go there, so the money comes back into the pathology here.’ And it’s worked well. (APN 1)
    One of the primary financial challenges discussed was the inadequacy of bulk billing services. GP 2 noted, “it’s hard to do in a 10-min consult … and bulkbilling doesn’t cover the cost of the consult… Now, virtually no GPs bulkbill. The MBS [Medical Benefits Schedule] rebate just does not cover the cost of a consult. So it then means that the hospital outpatients clinic which are generally bulkbilled are the only free option.… that may be less of an incentive to see the GP because they’re, yeah, because it costs.”
    The cost of medical tests and timely access also emerged as a concern. APN 2 emphasised the need for solutions that allow patients to access tests without incurring significant expenses: “So that would be something to kind of work out where patients could go that they’re not paying a lot of money for all of these tests, and that they can get quick access to them I guess, or timely access… I guess that paying for – the fact that you don’t have to pay is like another facilitator to using the hospital.”
    Vaccinations represented another substantial expense for survivors. APN 1 expressed frustration over the costs associated with vaccinations, stating, “I still cannot believe we’re paying for them…. patients have to pay for their vaccinations. We’re talking a measly $2,000. It’s primary health care. Minimisation of hospitalisation. COVID should have told us that more than anything. Let’s vaccinate. Let’s provide them a cost of vaccination. You do it for splenectomy patients and solid transplant, why can’t we [BMT patients]?”.
  - The availability of community care
    Access to community care and the availability of GPs emerged as significant challenges in ensuring equitable long-term follow-up for BMT survivors.
    GPs are very busy and fitting these patients in and – it would probably be more than the usual 10 or 15 minute appointment. So I guess that’s a potential barrier from the GP point of view. (BMT Specialist 2)
    Other participants noted how the organisation of general practices and the instability in general practice staffing impacted upon patient’s engagement with community care. APN 1 highlighted that many patients struggle to find a consistent GP due to high turnover rates: “…a bigger issue in this day and age too, is with the way the GP practices are organised… As many patients will say, unless they’ve secured a really good one and they’ve had one most of their life, the ability to find a GP that stays in a practice… A lot of them get quite distressed if they’ve been with a long-term GP and they’re going, ‘Our GP’s leaving, don’t know what to do.’ It does leave them in a hard place, it really does.”
  - Access to long-term Care
    Ensuring equitable access to long-term follow-up care for BMT survivors was identified as a critical issue by participants. They highlighted the need for all patients to have access to necessary care without financial or logistical barriers, emphasising that follow-up care should be available and easily accessible to everyone.
    Just making sure every patient gets the right care, that’s the ultimate…. any patient who wants follow-up care should be able to get and get it easily and get it without cost. (APN 4)
    However, logistical challenges in coordinating long-term follow-up and a lack of dedicated administrative support were significant barriers. Participants described how coordinating patient reminders, organising appointments, and accurate data entry and management would benefit both patients and clinicians.
    To identify people who are at one year, two years, five years, 10 years, 15 years. to have a mechanism for reminding them to come in, to saying to them, ‘You need to get the following test done,’ to collect the results of those tests, to put it in the information systems. (BMT Specialist 3)
    And the other part of that job is booking so many tests and so many – and we try and be good and do them all on the same day. So, the nurse spends half the time booking tests and then following up tests, and it’s very time-consuming. (APN 4)
    Current systems were noted to often fall short in providing such support. BMT Specialist 3 noted that while doctors and APNs attempt these tasks, they are not always executed effectively: “That’s the kind of stuff, for example, that doctors do appallingly badly, nurse practitioners do better but still not brilliantly. But that very well-coordinated groups of administrators and data managers could do well, but they currently don’t.”
    There was general agreement that limited human and material resources created barriers to effective long-term follow-up regardless of location. Hospital clinics were described as being “for people to be diagnosed, receive urgent treatment, be looked after. Not for long-term follow-up of people who are well” (BMT Specialist 4). BMT Specialist 3 observed that “there needs to be adequate resourcing both in terms of space, in terms of money, in terms of educational resources, and in terms of staff.” The nurse participants echoed this sentiment. APN 3 identified how “we could employ a nurse, if we had any flipping money, and then she could coordinate the tests, she could monitor quality of life, things like that….” Similarly, APN 1 observed that “It’s really hard to get a clinic space just to review these patients. I’m kind of just – I’ll jump into an office of someone who’s away…It’s hard.”

## Discussion

The findings of this study emphasise the need for accessible, flexible, and patient-centred models of care developed in collaboration with primary healthcare providers to effectively manage the significant burden and complexity of survivorship [19]. Comprehensive education and health promotion should be prioritised, including tailored workshops, self-management programs, and psychoeducational resources to empower survivors in managing long-term and late side effects and their ‘new normal.’ Integration of tertiary and primary care expertise is critical, with shared care models enabling coordinated follow-up plans that leverage specialist knowledge while ensuring continuity through general practice.

Routine holistic needs assessments should be embedded in survivorship care to systematically identify physical, psychosocial, and practical challenges. The adoption of telehealth can enhance access for rural and mobility-limited survivors. Digital health platforms such as My Health Record in Australia can improve care co-ordination and patient self-efficacy. To address financial inequities, multi-level strategies are required including systemic reforms (e.g. insurance coverage), health system interventions (e.g. financial navigation programs), and community support networks to mitigate out-of-pocket costs for medications, travel and lost wages [20] may address equity and access challenges.

Our study reinforces previously identified challenges in providing equitable and holistic long-term care, emphasising the importance of tailoring care to individual survivor needs, circumstances, and preferences [21]. Barriers such as the complexity of follow-up care and difficulties in providing comprehensive education and support align with literature describing how health systems that focus on biomedical approaches and standardisation of care compromise individualised care [21]. Additionally, significant challenges in providing comprehensive education to BMT survivors, particularly regarding timing and content delivery, were identified. These findings align with Rozwadowski et al.’s [11] work on promoting health and well-being in BMT patients and their caregivers. The need for tailored education strategies along the survivorship trajectory is evident.

Adherence to long-term follow-up guidelines emerged as a significant challenge in our study, with various factors influencing survivors’ engagement with ongoing care. This aligns with literature on healthcare utilisation by long-term survivors of BMT [22, 23]. Equity and access challenges, including geographical barriers, financial burdens, and limited community-based care availability, reflect broader cancer survivorship care issues. Despite progress, many recommendations from earlier reports remain unimplemented [24], with substantial obstacles in delivering standardised physical and psychosocial care services. Our study emphasises the need for flexible care models, including telehealth, shared care, and digital health platforms (made available to the survivor and all HCPs involved in their care), to address these challenges. The financial burdens identified in our study, particularly related to travel costs and out-of-pocket expenses for tests and vaccinations, support the need for policy interventions to address cost barriers [25].

## Limitations

This study has several limitations. The relatively small sample size may not fully capture the diverse experiences of all clinicians involved in BMT survivor care, particularly regarding underserved groups such as First Nations peoples, culturally and linguistically diverse communities, and children/adolescents and young adults (AYA). Conducting the study in one Australian state limits the generalisability of findings to other settings and countries. Additionally, the experiences of clinicians caring for longer-term survivors, especially those transitioning from AYA to adult care, may not be adequately addressed. This is significant as these cohorts can face challenges due to changes in care or loss of continuity. Lastly, with improved survival rates, particularly among paediatric and AYA patients, the long-term care needs and experiences of clinicians managing these survivors may not be fully represented in our findings.

## Conclusion

The study findings aligns with previous Australian research on the complex care needs of BMT survivors [2, 3, 3, 4, 26–28], highlighting critical implications for improving care and equity. However, our findings emphasize the need for patient-centred multidisciplinary models of survivorship care that integrate both tertiary and primary healthcare expertise. To address the multifaceted challenges faced by BMT survivors, we recommend the routine use of holistic needs assessments for survivors and caregivers, which can systematically identify and address physical, psychosocial, and practical concerns [29]. Flexible care models—including shared care between transplant centres and local providers, telehealth, and digital health platforms such as My Health Record—can improve access, continuity, and patient engagement, particularly for those in rural or underserved areas. Evidence supports the safety and acceptability of shared care models, which can reduce patient burden and maintain quality of life [30]. Comprehensive education and health promotion that is tailored to individual needs and delivered across the survivorship trajectory are essential for empowering survivors in self-management and informed decision-making. Finally, addressing financial barriers through integrated financial counselling, navigation services, and advocacy for policy reforms is crucial to ensure equitable access to care and support long-term health and wellbeing for all BMT survivors. Implementing these evidence-based, multifaceted strategies can enhance care delivery and improve outcomes and ultimately the quality of life for BMT survivors.

## Data Availability

No datasets were generated or analysed during the current study.
